# Comparative Cancer Genetics and Veterinary Therapeutics in Dogs and Cats: A Species-Aware Framework for Comparative Oncology

**DOI:** 10.3390/life16030430

**Published:** 2026-03-06

**Authors:** Sangjin Ahn, Jang-Hyuk Yun

**Affiliations:** 1 College of Veterinary Medicine and Institute of Veterinary Science, Kangwon National University, Chuncheon 24341, Republic of Korea; wildlife@kangwon.ac.kr; 2 Gangwon Wildlife Medical Rescue Center, Chuncheon 24341, Republic of Korea

**Keywords:** anticancer therapy, companion animals, comparative oncology, drug delivery systems, immunotherapy, nanoparticles, pharmacokinetics, precision medicine, spontaneous tumors, translational pharmaceutics

## Abstract

Cancer remains one of the leading causes of death worldwide, affecting not only humans but also companion animals such as dogs and cats. Although traditional rodent models have long served as the foundation of preclinical oncology research, their limited ability to replicate the complexity of spontaneous human cancers has driven interest in comparative oncology. Dogs and cats develop naturally occurring tumors that closely resemble human malignancies in histopathology, molecular alterations, tumor microenvironments, and treatment response. These species also share exposure to environmental carcinogens and demonstrate conserved pharmacokinetic and pharmacodynamic (PK/PD) profiles of several chemotherapeutic agents. This review provides a comprehensive comparison of cancer epidemiology, tumor biology, pharmacologic treatment modalities, drug formulation challenges, and regulatory considerations in humans, dogs, and cats. Key molecular targets such as TP53, HER2, EGFR, and PD-L1 exhibit cross-species conservation, supporting the relevance of companion animals in biomarker discovery and drug screening. However, interspecies differences in drug metabolism, enzyme expression (e.g., CYP450), and drug tolerability underscore the importance of species-specific dosing strategies and therapeutic drug monitoring. Advanced delivery platforms—including liposomes, nanoparticles, and antibody–drug conjugates—have also been successfully translated into veterinary oncology. Comparative analyses across humans and companion animals may inform biomarker prioritization, PK/PD modeling, and formulation design; however, species-specific differences require cautious interpretation and veterinary-centered dosing and safety considerations. Companion animals serve not only as valuable models for cancer research but also as direct beneficiaries of precision oncology. Future work should prioritize harmonized veterinary trial designs and improved cross-species data standards, with translational applications considered as a downstream benefit.

## 1. Introduction

Cancer represents a significant global health challenge, ranking as the second leading cause of death worldwide. According to the most recent GLOBOCAN 2022 estimates, nearly 20 million new cancer cases and approximately 9.7 million cancer-related deaths occurred worldwide in 2022 [1,2]. Although oncology research primarily focuses on human disease, cancer is also a leading cause of death in companion animals, particularly aging dogs and cats. In elderly dogs, neoplasia accounts for nearly half of all deaths, highlighting cancer as a major age-associated cause of mortality [3]. Although cancer prevalence differs between species, mammary carcinoma and lymphoma are among the most commonly diagnosed malignant tumours in cats [4,5].

Despite the extensive use of traditional preclinical models such as murine xenografts and genetically engineered mice, these systems present substantial translational limitations. Tumors in rodent models are typically induced or genetically manipulated, limiting their ability to fully replicate the complexity, heterogeneity, and tumor microenvironment of spontaneously arising human cancers. Moreover, significant differences in immune responses, metabolic pathways, and drug clearance between rodents and humans reduce the predictive accuracy of these models in drug development, often leading to poor clinical translatability and high attrition rates in late-stage trials [6,7].

In contrast, companion animals such as dogs and cats develop naturally occurring, histopathologically and genetically diverse tumors that closely mimic human cancers in disease progression, metastasis, and therapeutic response. These species share not only key oncogenic pathways—such as mutations in TP53, PTEN, and HER2—but also comparable tumor microenvironments and physiological conditions. Importantly, they live in the same environments as humans, experience similar exposures to carcinogens, and develop cancer naturally, without experimental induction or genetic engineering. The relatively short lifespans of dogs and cats make them ideal candidates for accelerated clinical evaluation of cancer therapies, allowing longitudinal studies within a compressed timeframe and yielding clinically relevant insights into therapeutic efficacy and toxicity [8,9].

Notably, specific canine and feline cancers have emerged as relevant translational models for human malignancies. Canine oral melanoma shares important biological and immunologic characteristics with human mucosal melanoma, while feline oral squamous cell carcinoma demonstrates comparable histologic and molecular features to its human counterpart, supporting their value in comparative oncology research [10,11].

This review synthesizes canine and feline cancer genetics and veterinary therapeutic strategies, with particular emphasis on epidemiology, tumor biology, and pharmacologic considerations. By clarifying shared and divergent mechanisms across dogs and cats, we aim to strengthen species-aware veterinary oncology frameworks while identifying areas where comparative observations may be informative beyond veterinary medicine.

## 2. Comparative Cancer Epidemiology

Cancer remains a leading cause of morbidity and mortality in both humans and companion animals, particularly dogs and cats, underscoring the need for cross-species oncologic research. Epidemiological data reveal notable parallels, as well as species-specific distinctions, that underscore the translational value of companion animals in cancer drug development.

**Figure 1:**
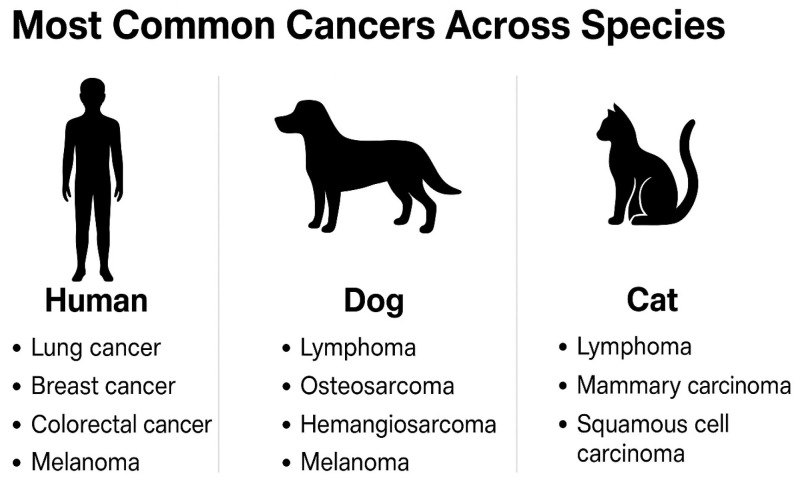
Most common cancer types across species. Comparative summary of frequently diagnosed malignancies in humans, dogs, and cats. In humans, breast, lung, and colorectal cancers are most prevalent, along with melanoma. In dogs, the leading tumor types include lymphoma, osteosarcoma, hemangiosarcoma, and melanoma. Cats most commonly develop lymphoma, mammary carcinoma, and squamous cell carcinoma. This cross-species overview highlights shared oncologic patterns and supports the use of companion animals in translational cancer research.

In humans, the lifetime risk of developing cancer is approximately 40% in Western countries. Global cancer statistics highlight a substantial worldwide burden, with a considerable lifetime probability of developing cancer [1,12]. The most common tumor types observed across the species are summarized in Figure 1. Among canine populations, cancer is also highly prevalent, with an estimated lifetime incidence of about 25–33%, increasing to nearly 50% in dogs over 10 years of age [13,14]. Malignancies are a leading cause of death in dogs, particularly in medium-to-large breeds. The most common tumor types reported in dogs include lymphoma, osteosarcoma, hemangiosarcoma, and melanoma, among others. Osteosarcoma occurs substantially more frequently in dogs than in humans, supporting the use of dogs as a robust comparative model for pediatric bone cancer. Genetic predisposition is pronounced in certain breeds; for example, Rottweilers and Great Danes have a high risk for osteosarcoma, while Golden Retrievers and Boxers are predisposed to lymphoma and hemangiosarcoma [14,15,16].

Cancer is a significant cause of death in cats, although reported incidence appears lower than in dogs [17,18]. Lymphoma—often associated with feline leukemia virus (FeLV) or feline immunodeficiency virus (FIV)—mammary carcinoma, and squamous cell carcinoma are the most frequently diagnosed tumors [19,20]. Notably, early ovariohysterectomy in cats reduces the risk of mammary carcinoma by over 90% when performed before six months of age [21]. Lymphoma in cats is strongly associated with viral oncogenesis, particularly FeLV and FIV infections [22].

Shared environmental exposure further supports the translational relevance of companion animals. Humans, dogs, and cats share living spaces and are commonly exposed to carcinogens such as tobacco smoke, air pollutants, and household chemicals [23,24,25]. Thus, companion animals serve as valuable sentinels for environmental cancer risks relevant to human health.

Genetic factors in dogs are amplified through selective breeding. Many canine cancers exhibit molecular alterations similar to those observed in human cancers, including mutations in TP53, RB1, PTEN, and KIT [16,26]. These shared oncogenic pathways enhance translational impact in canine models. Human–canine comparisons are particularly informative in osteosarcoma: dogs have a much higher incidence and homologous tumor biology, promoting insights into bone-targeted therapies [27,28].

Several reviews, such as those by Paoloni and Khanna [29] and Khanna et al. [30], have emphasized the value of spontaneous cancers in pet dogs as translational models for evaluating novel therapeutics. These naturally occurring models offer a unique opportunity to bridge the translational gap between preclinical rodent studies and human clinical trials by enabling the integrated assessment of safety, pharmacokinetics, pharmacodynamics, and therapeutic efficacy in a physiologically relevant context [31,32].

In summary, the lifetime risks of cancer, shared environmental exposures, spontaneous tumor development, and overlapping molecular pathways in humans, dogs, and cats provide a biologically relevant and powerful foundation for comparative oncology. Collectively, these interspecies synergies support the integration of companion animal clinical trials into drug development pipelines, facilitating more robust target validation, formulation optimization, PK/PD modeling, and accelerated translation for human use.

A comparative summary of major cancer types, etiological features, and translational relevance across humans, dogs, and cats is presented in Table 1.

## 3. Tumor Biology and Molecular Markers

### 3.1. Shared Molecular Pathways

Tumorigenesis is driven by a range of conserved molecular events across species, with numerous genetic, environmental, and immunological factors contributing to the development and progression of cancer in humans, dogs, and cats. Among the most frequently altered genes is TP53, a tumor suppressor gene that encodes the p53 protein, which regulates cell cycle arrest, DNA repair, and apoptosis [33,34]. In humans, mutations in TP53 occur in over half of all cancers and are strongly associated with a poor prognosis [35,36,37]. Similarly, TP53 mutations are frequently observed in canine osteosarcoma, mammary gland tumors, and lymphoma, and have also been reported in feline mammary carcinomas, suggesting cross-species conservation of this critical tumor suppressor pathway [38,39,40,41].

Receptor tyrosine kinases, such as HER2 and EGFR, also play significant roles in oncogenesis. HER2 amplification is well characterized in human breast cancer and has been therapeutically targeted by monoclonal antibodies such as trastuzumab [42,43]. In companion animals, HER2 overexpression has been reported in canine and feline mammary carcinomas, indicating molecular parallels in tumorigenic signaling cascades [44,45]. Additionally, EGFR is overexpressed in canine transitional cell carcinoma of the bladder and other tumors, further supporting its translational relevance as a therapeutic target across species [46].

Mutations in the BRAF oncogene represent another important example of cross-species molecular relevance in comparative oncology. In humans, activating BRAF mutations—most notably the V600E substitution—have been identified in multiple malignancies, including melanoma, colorectal cancer, and urothelial carcinoma, and are associated with aberrant activation of the MAPK signaling pathway [47,48,49,50]. In canine oncology, a homologous BRAF mutation (V595E) has been detected in a high proportion of invasive urothelial carcinomas and has also been reported in canine prostatic carcinomas [51,52]. This conserved mutation not only enhances the diagnostic utility of molecular testing in dogs but also underscores the translational value of spontaneous canine tumors as models for BRAF-driven human cancers. The inclusion of BRAF further strengthens the comparative molecular framework of this review.

Alterations in the RAS/PI3K/AKT signaling axis further illustrate conserved oncogenic mechanisms across species. Activating mutations in KRAS and components of the PI3K pathway are frequently observed in human colorectal, pancreatic, and lung cancers and contribute to sustained proliferative signaling [53,54]. Comparable dysregulation of the PI3K/AKT pathway has been reported in canine mammary tumors, supporting conservation of mitogenic and survival signaling cascades across species [55].

A recent large-scale transcriptomic analysis revealed that osteosarcomas in both dogs and humans share conserved tumor microenvironment (TME) subtypes, including the immune-enriched, matrix-dominant, and immune-desert phenotypes [56]. The TME is a critical determinant of cancer progression and therapeutic response, comprising stromal fibroblasts, immune cells, endothelial components, and the extracellular matrix, all of which interact dynamically with tumor cells. These conserved microenvironmental profiles influence treatment outcomes and support the rationale for translational studies targeting immune modulation and stromal remodeling [56].

**Figure 2:**
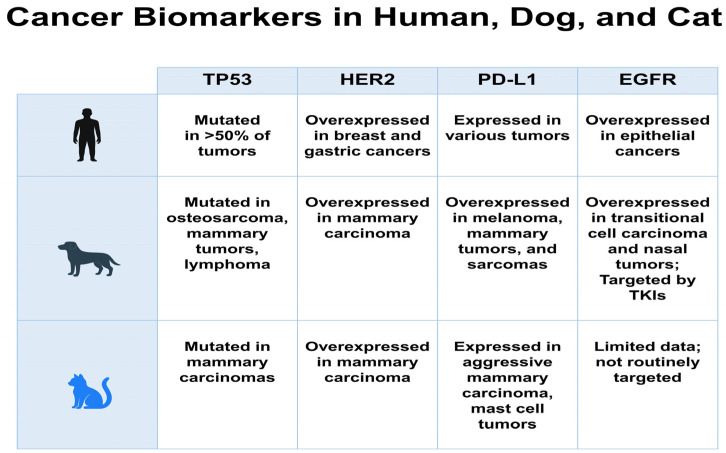
Comparative overview of key cancer biomarkers (TP53, HER2, PD-L1, EGFR) across humans, dogs, and cats. Expression and mutation patterns are summarized to illustrate cross-species similarities and differences with translational relevance in oncology.

Biomarkers such as PD-L1 are increasingly used to guide precision oncology and immunotherapy. In humans, PD-L1 expression levels correlate with the response to checkpoint inhibitors in cancers such as melanoma and non-small-cell lung carcinoma [57,58]. Notably, spontaneous tumors in dogs also frequently express PD-L1, particularly oral malignant melanoma, mammary carcinoma, and certain sarcomas [59]. Therapeutic trials using canine-specific anti-PD-L1 antibodies have shown promising results in enhancing survival and promoting immune activation [60,61]. While data in cats remains limited, PD-L1 expression has been detected in some cases of feline mammary carcinomas and mast cell tumors, particularly in those exhibiting aggressive histological features or HER2 positivity [62,63]. A detailed comparative summary of key molecular markers and therapeutic targets across species is presented in Table 2. To enhance visual comprehension, Figure 2 provides a schematic overview of the key biomarkers and their translational relevance.

### 3.2. Species-Specific Differences in Carcinogenesis and Tumor Evolution

While substantial molecular similarities exist across humans and companion animals, biologically and clinically significant interspecies differences must also be emphasized. In humans, carcinogenesis is typically driven by the gradual accumulation of somatic mutations associated with aging, environmental carcinogens, lifestyle factors, and chronic inflammation. Multistep clonal evolution, accumulation of driver mutations, and genomic instability represent dominant mechanisms in many human malignancies.

In contrast, canine cancers frequently demonstrate strong breed-associated genetic predispositions resulting from selective breeding practices. Certain large and giant breeds exhibit disproportionately high incidences of osteosarcoma and hemangiosarcoma, reflecting inherited genomic risk patterns that differ from the broader genetic heterogeneity observed in human populations [14,15]. This breed-driven genetic architecture may influence tumor onset, progression kinetics, and therapeutic responsiveness.

Feline oncogenesis displays additional distinctive characteristics, particularly in lymphoma. Viral etiologies such as feline leukemia virus (FeLV) and feline immunodeficiency virus (FIV) play a significant role in tumor initiation and progression [22]. In addition to these etiologic drivers, molecular characterization of feline cancers has historically been more limited than in dogs; however, recent large-scale oncogenomic efforts have substantially expanded the available datasets. Comprehensive genomic profiling of domestic cats has now provided expanded resolution of recurrent tumor-associated alterations [64], facilitating more systematic comparative analyses. Viral-mediated insertional mutagenesis and immune dysregulation are more prominent in feline cancers than in canine and most human malignancies. Moreover, feline mammary carcinoma often exhibits more aggressive biological behavior compared to canine mammary tumors and frequently resembles high-grade, hormone-independent human triple-negative breast cancer [65,66].

Differences in tumor microenvironment composition, immune surveillance mechanisms, checkpoint regulation dynamics, and stromal interactions further contribute to species-specific patterns of tumor progression and therapeutic response. These distinctions underscore the importance of interpreting comparative oncology data within a species-aware biological framework rather than assuming complete mechanistic equivalence.

Overall, although substantial molecular similarities exist across species, meaningful biological differences in carcinogenesis and tumor evolution must also be considered. Importantly, recognizing both shared and divergent mechanisms strengthens the translational validity of comparative oncology models. Furthermore, they facilitate the development of more effective pharmaceutical interventions by enabling early biomarker validation, improving pharmacokinetic/pharmacodynamic (PK/PD) modeling, and identifying shared molecular targets in naturally occurring disease contexts.

While these species-level contrasts highlight meaningful biological divergence from human oncogenesis, important differences also exist between dogs and cats themselves. A direct dog–cat comparison is therefore essential to clarify when each species provides distinct biological and therapeutic insight. The following section focuses specifically on these intra-companion animal contrasts.

### 3.3. Dog–Cat Contrasts: Tumorigenesis, Treatment Constraints, and Model Selection

#### 3.3.1. Etiology and Genetic Architecture

Although dogs and cats share conserved oncogenic pathways, the upstream determinants of cancer risk and the genomic architecture shaping tumorigenesis differ substantially between the two species. In dogs, selective breeding has generated relatively closed genetic populations, resulting in strong breed-associated predispositions for specific malignancies [14,67]. This breed-structured risk enables the study of genetically enriched tumor subtypes within defined backgrounds, facilitating susceptibility locus identification [68,69,70,71]. However, such genetic concentration may also limit broader generalizability across heterogeneous populations.

In cats, breed effects are less dominant in many clinical contexts, and distinct etiologic drivers are more prominent. Lymphoma biology and hormonally influenced risk in mammary carcinoma represent key biological features [72,73]. Feline mammary carcinoma frequently exhibits aggressive clinical behavior and molecular phenotypes that resemble high-grade epithelial malignancies [74]. These distinctions indicate that while canine cancers often provide genetically enriched models, feline cancers offer unique insights into virus-associated tumor evolution and aggressive epithelial disease biology.

#### 3.3.2. Tumor Spectrum and Clinical Course

The relative frequency and clinical course of major tumor types differ meaningfully between dogs and cats. In dogs, lymphoma, osteosarcoma, hemangiosarcoma, melanoma, and urothelial carcinoma are commonly encountered, with some entities occurring at substantially higher rates than in cats [75]. Breed size and lifespan further influence age at cancer onset across canine populations [76,77].

In contrast, cats most commonly present with lymphoma, mammary carcinoma, and squamous cell carcinoma [20]. Feline mammary carcinoma is frequently aggressive, and lymphoma may show etiologic heterogeneity influenced by viral status [22,78]. These tumor-spectrum differences are central to species selection in comparative oncology, as they determine which biological and therapeutic questions can be addressed most effectively within each species.

#### 3.3.3. Treatment Landscape and Tolerability

While dogs and cats are treated with broadly similar classes of chemotherapeutic agents, differences in routine clinical implementation and tolerability must be emphasized. In dogs, certain targeted therapies and multicenter clinical trial infrastructures are more established in many regions, enabling longitudinal monitoring and standardized dosing strategies [79,80].

In cats, treatment may be constrained by species-specific toxicity vulnerabilities [81]. Evidence supporting some targeted and immunotherapeutic approaches remains comparatively limited in feline oncology [82]. These differences underscore the necessity of presenting therapeutic evidence separately for each species and clearly distinguishing established veterinary protocols from extrapolated or emerging strategies.

#### 3.3.4. Pharmacokinetics, Pharmacodynamics, and Metabolic Constraints

Species-specific pharmacokinetic and metabolic differences represent a fundamental contrast between dogs and cats. Cats exhibit reduced capacity for certain phase II conjugation pathways, including glucuronidation for relevant substrates, which can predispose them to drug accumulation and toxicity [83]. This metabolic limitation directly influences drug selection, dosing intervals, and the need for cautious escalation strategies in feline oncology.

Dogs do not share this characteristic metabolic constraint but may demonstrate inter-breed and inter-individual variability in drug-metabolizing enzymes, contributing to differences in systemic exposure and toxicity profiles [84]. Consequently, canine oncology often highlights variability across breeds and body sizes, whereas feline oncology emphasizes categorical metabolic considerations that require strict species-specific dosing logic and careful monitoring.

#### 3.3.5. Model Selection Considerations

Taken together, these interspecies contrasts suggest that model selection should be tumor-type and research-question driven. Dogs may be particularly informative for high-incidence sarcomas, genetically enriched tumor subtypes, and studies requiring scalable clinical trial infrastructure. Cats may provide distinctive value in virus-associated malignancies, aggressive mammary carcinoma, and research contexts where metabolic constraints and tolerability considerations shape therapeutic feasibility.

Recognizing both shared mechanisms and species-specific divergences strengthens the biological and clinical validity of comparative oncology. A species-aware framework prevents overgeneralization and ensures that veterinary therapeutic strategies remain grounded in evidence specific to each companion animal population. Table 3 provides a structured comparative overview of key interspecies contrasts relevant to oncology research and translational model selection.

## 4. Pharmacological Treatment Modalities

In this review, we compare pharmacological approaches to cancer treatment in humans, dogs, and cats, focusing on major drug classes, regulatory approval versus off-label usage, and interspecies pharmacokinetic/pharmacodynamic (PK/PD) differences, including illustrative case studies.

### 4.1. Comparison of Drug Classes and Approval Status

Alkylating agents, platinum compounds (e.g., carboplatin), and anthracyclines (e.g., doxorubicin) are among the most widely used chemotherapeutic drugs in human and veterinary medicine. These agents are commonly administered off-label to dogs and cats; however, their efficacy and safety profiles have been well characterized in canine osteosarcoma, lymphoma, and mammary carcinoma [85,86,87,88]. Additionally, tyrosine kinase inhibitors (TKIs) are a class of targeted therapies with significant translational relevance [89,90]. Toceranib phosphate (Palladia) is currently the only TKI officially approved for veterinary use—specifically for canine mast cell tumors—by regulatory authorities, including the Food and Drug Administration (FDA) and the European Medicines Agency (EMA) [91,92]. Its standard dosing regimen of 3.25 mg/kg, administered orally every other day, has demonstrated clinical activity with an acceptable safety profile [91,92,93]. Although TKIs such as masitinib are used off-label in feline patients, no TKIs have yet achieved formal approval for cats [90,94,95,96].

### 4.2. Interspecies Pharmacokinetic and Pharmacodynamic Differences

Differences in drug-metabolizing enzymes across species, such as those in the cytochrome P450 family, influence drug clearance, half-life, and toxicity. In small-breed dogs, body surface area-based dosing of doxorubicin has been associated with relatively higher systemic exposure and increased risk of myelosuppression compared to larger dogs, although definitive pharmacokinetic evidence remains limited [97]. Carboplatin exhibits relatively consistent pharmacokinetic behavior across species, although inter-individual variability has been reported in dogs; some studies suggest more predictable exposure when dosing is adjusted appropriately [88,98]. A recent study highlighted that body surface area dosing alone may not sufficiently predict toxicity, advocating for physiologically informed dosing methods based on glomerular filtration rate and targeted AUC values [98,99].

Beyond pharmacokinetic variability, fundamental interspecies differences in drug metabolism mechanisms must be considered. Cats exhibit reduced glucuronidation capacity due to limited expression of specific UDP-glucuronosyltransferase (UGT) isoforms, predisposing them to drug accumulation and toxicity when exposed to agents primarily eliminated through phase II conjugation pathways [83]. This reduced conjugation capacity represents one of the most clinically significant metabolic divergences among companion animals and has important implications for chemotherapeutic selection and dose optimization in feline oncology.

In dogs, polymorphisms in cytochrome P450 (CYP450) genes have been identified across multiple breeds and may contribute to inter-breed variability in drug metabolism and systemic exposure [100]. Polymorphisms and differential enzyme activity may influence both efficacy and toxicity profiles. In contrast, human drug metabolism is influenced by broader genetic polymorphisms, comorbid conditions, and polypharmacy, creating additional variability in treatment response.

These interspecies metabolic distinctions highlight the limitations of direct dose extrapolation based solely on body surface area and reinforce the need for physiologically informed dosing strategies supported by therapeutic drug monitoring. A practical example of species-specific pharmacokinetic optimization is provided by toceranib therapy in dogs.

Toceranib has an elimination half-life of approximately 17 h when administered intravenously and extends to approximately 31 h after oral administration in dogs. Oral bioavailability was high (~77%), and pharmacokinetic parameters remained consistent even with chronic dosing every other day [92,101]. Lowering the dose to 2.4–2.9 mg/kg has produced clinically relevant blood concentrations (100–120 ng/mL at 6–8 h post-dose) while reducing adverse events compared to the approved 3.25 mg/kg regimen [102].

### 4.3. Representative Case Studies

Several pharmacokinetic/pharmacodynamic (PK/PD) studies have demonstrated the need for species-specific dosing strategies. Co-administration of doxorubicin and valproic acid in dogs revealed no significant PK interaction; however, valproic acid-induced histone hyperacetylation, offering a pharmacodynamic rationale for combination regimens [103]. A Phase I study of sorafenib (a multi-targeted TKI) administered at 3 mg/kg in dogs demonstrated good tolerability, laying the groundwork for oral dosing schedules in veterinary patients [104]. In canine mammary carcinoma, carboplatin dosed at 300 mg/m^2^, either alone or in combination with metronomic cyclophosphamide, showed predictable pharmacokinetics. The combination regimen demonstrated acceptable tolerability and suggested potential survival benefits, supporting further optimization of combination therapy strategies [88]. Combination strategies incorporating platinum-based agents and tyrosine kinase inhibitors have been explored in canine oncology, demonstrating acceptable tolerability and suggesting potential clinical benefit, thereby supporting the optimization of therapeutic regimens through improved cross-species PK/PD understanding [88,92,105,106].

### 4.4. Summary and Implications for Comparative Oncology

Although humans, dogs, and cats are frequently treated with similar chemotherapeutic agents, fundamental interspecies differences in metabolic pathways and drug tolerance necessitate species-specific therapeutic strategies. However, significant interspecies differences in metabolism, distribution, clearance, and toxicity necessitate individualized PK/PD dosing protocols. Body surface area-based dosing provides a rough guideline, but therapeutic drug monitoring (TDM) based on AUC or plasma concentrations provides a more precise method for optimizing efficacy while minimizing adverse effects [99,107,108]. The case of toceranib illustrates that a lower tailored dose can sustain pharmacodynamic activity with improved tolerance [92,102]. Therefore, companion animal cancers represent spontaneous, translational models for human oncology, particularly when supported by rigorous PK/PD studies and dose optimization strategies.

## 5. Pharmaceutical Formulations and Drug Delivery Strategies

Effective cancer chemotherapy in companion animals requires careful consideration of both the route of administration and formulation used, as each species presents unique pharmacological and practical challenges. Oral administration is convenient and enables chronic treatment regimens; however, factors such as palatability, variable absorption, and owner compliance can compromise therapeutic efficacy in dogs and cats. Injectables—whether administered intravenously, intramuscularly, or subcutaneously—ensure precise dosing but often require veterinary visits and may lead to stress or poor compliance.

Advanced drug delivery systems such as nanoparticles, liposomes, and antibody–drug conjugates (ADCs) have been extensively studied in veterinary oncology to improve tumor targeting, reduce systemic toxicity, and address formulation limitations [109,110,111]. Liposomal formulations of doxorubicin (e.g., Doxil/Caelyx) demonstrate reduced cardiotoxicity compared to free drugs in dogs and cats, making them preferable for patients with pre-existing cardiac conditions [112,113]. Polymer-based nanoparticles encapsulating agents such as cisplatin and paclitaxel, as well as hyaluronic acid-based carriers, have shown promising preclinical results in naturally occurring canine tumors, with several nanoformulations currently under evaluation in clinical trials [114,115,116]. A recent study validated folate-targeted liposomes loaded with panobinostat in canine B-cell lymphoma models, demonstrating enhanced tumor uptake and providing comparative advantages in translational oncology [117].

Antibody–drug conjugates are emerging as a promising class of therapeutics in oncology, with increasing interest in veterinary applications [118]. OS Therapies have reported (company data) the development of an HER2-targeted ADC for canine osteosarcoma, leveraging advanced linker technologies and payloads specifically adapted to canine biology. Moreover, a canine-specific monoclonal antibody against podoplanin (P38Bf) has undergone Phase I/II studies in dogs with spontaneous malignant melanoma, showing minimal toxicity and preliminary evidence of disease stabilization [119].

Despite these innovations, formulation challenges unique to veterinary medicine remain significant, such as palatability concerns for oral products, reliance on owner-administered dosing, and the need for easy-to-administer injectable drugs. Companion animals may refuse bitter-tasting oral medications, reducing their bioavailability and treatment adherence [120]. Injectable formulations must balance drug potency with an acceptable injection volume and frequency to ensure compliance and minimize handling stress for both animals and owners [121].

In summary, the development of pharmaceutical formulations for veterinary oncology must integrate advanced delivery technologies with the practical constraints inherent to animal use. Nanoparticles and liposomes have demonstrated tangible benefits in terms of toxicity reduction and targeted delivery, whereas ADCs hold translational promise for the treatment of naturally occurring cancers in pets. Simultaneously, oral formulations must address taste masking and ease of administration, while injectables must be formulated for minimal stress and maximum dosing accuracy. This dual focus—technological innovation anchored in animal welfare and owner compliance—will be critical in guiding the next generation of veterinary cancer therapeutics.

## 6. Comparative Oncology Infrastructure and Cross-Species Interpretation

Companion animals, particularly dogs and cats, provide clinically relevant settings for studying naturally occurring cancers within veterinary oncology. Unlike experimentally induced tumor models, spontaneous malignancies in companion animals develop within intact immune systems and real-world environmental contexts [122]. The primary value of comparative oncology therefore lies in strengthening evidence-based veterinary cancer care while enabling structured cross-species interpretation when biologically appropriate.

### 6.1. Veterinary Clinical Trial Infrastructure

Organized veterinary clinical trial networks, such as the Comparative Oncology Trials Consortium (COTC), provide multicenter platforms for evaluating therapeutic strategies in dogs with naturally occurring tumors [79]. These infrastructures facilitate standardized study design, pharmacokinetic and pharmacodynamic sampling, biological endpoint definition, and coordinated data collection across institutions [79].

Such frameworks improve reproducibility, enable longitudinal monitoring, and support rigorous assessment of therapeutic feasibility within veterinary populations. Importantly, these networks operate within veterinary regulatory and ethical standards, emphasizing owner consent, animal welfare, and clinically meaningful endpoints specific to companion animals [123].

### 6.2. Trial Feasibility and Endpoint Considerations in Companion Animals

Veterinary oncology trials offer practical advantages, including access to spontaneous tumors, defined staging systems, and real-world therapeutic monitoring. However, species-specific considerations must guide trial design. In dogs, breed-associated predispositions may influence case composition and genetic homogeneity [14]. In cats, metabolic constraints, tolerability limitations, and practical administration challenges may affect dosing strategies and protocol feasibility [81,82,83].

Careful endpoint selection—such as progression-free interval, quality-of-life metrics, and pharmacokinetic exposure—remains essential to ensure that trial outcomes are biologically and clinically meaningful within each species. A species-aware approach prevents overgeneralization and strengthens the validity of veterinary therapeutic conclusions.

### 6.3. Data Harmonization and Cross-Species Interpretation

Standardization of imaging protocols, tissue sampling procedures, biomarker assays, and pharmacokinetic methodologies enhances comparability across veterinary centers. Harmonized data collection improves statistical robustness and supports responsible comparative analyses.

When interpreted within clearly defined biological contexts, such harmonized datasets may inform broader cross-species research questions. However, extrapolation beyond veterinary oncology should remain tumor-specific, evidence-driven, and mindful of species-level biological divergence. Comparative oncology is most rigorous when it acknowledges both shared mechanisms and clinically meaningful differences.

## 7. Conclusions and Future Directions

The comparative study of cancer in humans, dogs, and cats highlights the profound pharmaceutical and translational potential of companion animal oncology [8]. Naturally occurring tumors in these species exhibit key histopathological, molecular, and pharmacokinetic similarities to human malignancies, offering a more clinically relevant and ethically acceptable alternative to traditional preclinical models [31]. The spontaneous nature of tumor development in companion animals—under similar environmental exposures and within intact immune systems—enables a more accurate recapitulation of human disease progression, therapeutic responses, and adverse event profiles. This comparative perspective allows researchers to more effectively evaluate drug efficacy, optimize pharmacokinetic/pharmacodynamic (PK/PD) relationships, and test delivery strategies in real-world biological contexts.

The integration of advanced drug delivery technologies, including liposomes and nanoparticles, into veterinary oncology has further reinforced the translational utility of these models [116]. These platforms improve drug targeting and reduce systemic toxicity while also providing insights into formulation challenges, such as palatability, treatment compliance, and dose scheduling, which are critical for therapeutic success in both animals and humans. Case studies involving toceranib, carboplatin, and doxorubicin demonstrate the importance of species-specific pharmacology and the need for individualized dosing strategies guided by PK/PD modeling and therapeutic drug monitoring [99,108,124,125].

As oncology continues to evolve toward precision medicine, the role of companion animals in drug development is expected to expand [8,126]. The incorporation of genomic, transcriptomic, and immunologic profiling in patients with veterinary cancer will facilitate more precise treatment matching, biomarker-driven patient selection, and the validation of novel therapeutic targets. Personalized oncology strategies—such as mutation-guided therapy, immune checkpoint blockade based on PD-L1 expression, and tumor microenvironment–targeted modulation—can be evaluated in real-world disease contexts through comparative oncology trials. These approaches will enable the earlier identification of responsive subpopulations, inform regulatory decisions, and accelerate therapeutic development in both human and veterinary oncology.

To fully realize the potential of comparative oncology, several key areas warrant future research [127,128,129]. First, expanding multi-institutional clinical trials involving spontaneous tumors in companion animals is essential to generate robust data on efficacy, safety, and long-term outcomes. Second, harmonizing regulatory frameworks between veterinary and human drug approval pathways is essential to streamline co-development processes and facilitate dual-species indications. Third, efforts should focus on standardizing imaging protocols, tissue sampling, and biomarker assays across species to ensure data comparability and quality. Finally, greater investment in veterinary-specific formulation development is required to address challenges related to compliance, stability, and targeted delivery.

In conclusion, the comparative study of cancer in dogs and cats provides a biologically robust and clinically relevant foundation for advancing veterinary oncology. Naturally occurring tumors in companion animals offer important opportunities to refine species-specific therapeutic strategies, optimize pharmacokinetic and pharmacodynamic approaches, and improve patient-centered cancer care. While comparative insights may inform broader oncologic research when interpreted cautiously and within appropriate biological contexts, the primary objective remains the advancement of evidence-based veterinary therapeutics.

## Figures and Tables

**Table 1:** Summary of major cancer types and epidemiological features across species.

Feature	Humans	Dogs	Cats
Lifetime Cancer Risk	~40%	~25–33%; >50% in dogs over 10 years	Estimated ~20–25%; increases with age
Most Common Cancers	Breast, lung, colorectal, and melanoma	Lymphoma, osteosarcoma, hemangiosarcoma, and melanoma	Lymphoma, mammary carcinoma, and squamous cell carcinoma
Viral Oncogenesis	HPV (cervical), EBV (lymphoma), and HBV/HCV (liver)	Rare	FeLV, FIV (notably in lymphoma)
Genetic Risk	BRCA1/2, TP53, and APC mutations	Breed-associated: TP53, RB1, PTEN	Limited data; hormonal and breed influence in mammary tumors suggested
Environmental Exposure	Tobacco, air pollution, and occupational and dietary carcinogens	Shared indoor environment with humans (e.g., tobacco smoke, pollutants)	Shared indoor environment (e.g., air pollutants, household chemicals)
Tumor Onset & Progression	Typically middle-aged to older adult onset	Most common in middle-aged and older dogs	Typically older age; younger onset in FeLV-associated lymphoma
Translational Relevance	Gold standard for translational research	High: spontaneous tumors, shared genetic pathways with humans	Moderate: relevant for viral and hormone-associated tumors

This comparative overview highlights key epidemiological features of cancer in humans, dogs, and cats—including lifetime risk, tumor types, viral involvement, genetic predisposition, environmental exposures, age of onset, and translational relevance. Such cross-species insights contribute to the development of more predictive preclinical models and inform comparative oncology strategies.

**Table 2:** Key molecular markers and therapeutic targets across species.

Marker	Humans	Dogs	Cats	Translational Relevance
TP53	Mutated in >50% of tumors	Mutated in osteosarcoma, mammary tumors, and lymphoma	Mutated in mammary carcinomas	Highly conserved tumor suppressor; model for drug resistance studies
HER2	Amplified in breast and gastric cancers	Overexpressed in mammary carcinoma	Overexpressed in mammary carcinoma	Target for trastuzumab-like therapies
PD-L1	Expressed in NSCLC and melanoma	Overexpressed in melanoma, mammary tumors, and sarcomas	Detected in aggressive or HER2-positive mammary carcinomas, mast cell tumors	Cross-species checkpoint inhibitor candidate
EGFR	Overexpressed in epithelial cancers	Overexpressed in transitional cell carcinoma and nasal tumors	Rarely reported	Target for tyrosine kinase inhibitors
BRAF	V600E mutation in melanoma, colorectal, and urothelial carcinoma	V595E mutation in urothelial and prostatic carcinoma	Rarely reported; limited molecular characterization	MAPK pathway target; diagnostic and translational relevance
KRAS/PI3K	KRAS mutations in colorectal, pancreatic, lung cancers; PIK3CA mutations common	PIK3CA mutations reported in mammary tumors; RAS pathway dysregulation observed in multiple malignancies	Limited data; emerging genomic studies ongoing	Conserved mitogenic and survival signaling pathways

Comparative summary of molecular targets relevant to tumor biology and therapy in humans, dogs, and cats.

**Table 3:** Comparative overview of dogs and cats in oncology research.

Domain	Dogs	Cats	Considerations for Comparative Research
Dominant tumor types	Lymphoma, osteosarcoma, hemangiosarcoma, melanoma, urothelial carcinoma	Lymphoma, mammary carcinoma, squamous cell carcinoma	Species selection should be tumor-type driven
Etiologic features	Strong breed-associated genetic predisposition	Viral influences in subsets of lymphoma	Dogs offer genetically enriched populations; cats offer virus-associated and aggressive epithelial contexts
Molecular landscape	Conserved tumor suppressor and RTK alterations; BRAF mutation reported in urothelial carcinoma	Expanding oncogenomic characterization; aggressive mammary carcinoma phenotypes	Present molecular evidence separately to avoid overgeneralization
Clinical course	High incidence of certain sarcomas; feasible longitudinal monitoring	Frequently aggressive mammary carcinoma; etiologic heterogeneity in lymphoma	Natural history differences influence endpoint design
Therapeutic landscape	Established multi-agent chemotherapy; some targeted therapies integrated into veterinary practice	Similar drug classes used; targeted/immunotherapy evidence more variable	Distinguish established protocols from emerging/off-label approaches
PK/PD considerations	Inter-breed metabolic variability	Reduced glucuronidation capacity for certain substrates	Species-specific dosing and monitoring are essential
Strengths as research models	High case numbers for specific tumors; genetic enrichment; trial feasibility	Unique etiologic contexts; aggressive epithelial tumor biology	Each species provides complementary insights
Limitations	Breed structure may limit generalizability	Lower incidence for some tumor types; dosing constraints	Interpret findings within species-aware biological context

## Data Availability

Data sharing is not applicable to this article as no new data were created or analyzed in this study.

## Abbreviations

The following abbreviations are used in this manuscript:

ADC	Antibody–Drug Conjugate
AUC	Area Under the Curve
BSA	Body Surface Area
COTC	Comparative Oncology Trials Consortium
CVM	Center for Veterinary Medicine
CYP450	Cytochrome P450
EGFR	Epidermal Growth Factor Receptor
EMA	European Medicines Agency
FDA	Food and Drug Administration
FeLV	Feline Leukemia Virus
FIV	Feline Immunodeficiency Virus
HER2	Human Epidermal Growth Factor Receptor 2
KIT	Receptor Tyrosine Kinase (c-Kit)
PD	Pharmacodynamics
PD-L1	Programmed Death-Ligand 1
PK	Pharmacokinetics
PTEN	Phosphatase and Tensin Homolog
RB1	Retinoblastoma 1
TDM	Therapeutic Drug Monitoring
TKIs	Tyrosine Kinase Inhibitors
TME	Tumor Microenvironment
TP53	Tumor Protein p53
VMP	Veterinary Medicines Product (EMA)
